# Yet another advantage of saline-immersion therapeutic endoscopy!

**DOI:** 10.1055/a-2334-0854

**Published:** 2024-06-25

**Authors:** Kosei Hashimoto, Hisashi Fukuda, Toshihiro Fujinuma, Edward J Despott, Hironori Yamamoto

**Affiliations:** 1 12838 Department of Medicine, Division of Gastroenterology, Jichi Medical University, Shimotsuke, Japan; 2 171090 Royal Free Unit for Endoscopy, The Royal Free Hospital, University College London Institute for Liver and Digestive Health, London, United Kingdom of Great Britain and Northern Ireland

Since its first description, saline-immersion therapeutic endoscopy (SITE) is being increasingly adopted to facilitate endoscopic submucosal dissection (ESD) 1 2 . SITE enhances access to submucosal pockets, and through buoyancy, obviates any need for traction. It augments visibility through magnification and elimination of smoke/debris, and its minimal distension of the lumen optimizes endoscopic maneuverability and patient comfort 1 3 . We report a further advantage of SITE.

An otherwise healthy 90-year-old man who had declined surgery underwent ESD of a large gastric tumor identified on computed tomography. The lesion consisted of a 60-mm Paris 0-Is component with a further 30-mm 0-IIa extension over the posterior wall of the lower gastric body ( Fig. 1 , Fig. 2 **)** . Lesion mobility and endoscopic ultrasound findings showed no signs of deep invasion. A fibrotic portion beneath the 0-Is area was dissected using the SITE-facilitated pocket-creation method (PCM) ESD ( Video 1 ) 4 . Thick perforating vessels were clipped to achieve a safe outcome. En bloc resection was achieved within 120 minutes. The large 0-Is component impeded safe passage through the esophagogastric junction and warranted snare division before retrieval. To maintain precise pathological submucosal integrity, only the mucosal portion was divided using a monopolar snare.

**Fig. 1:**
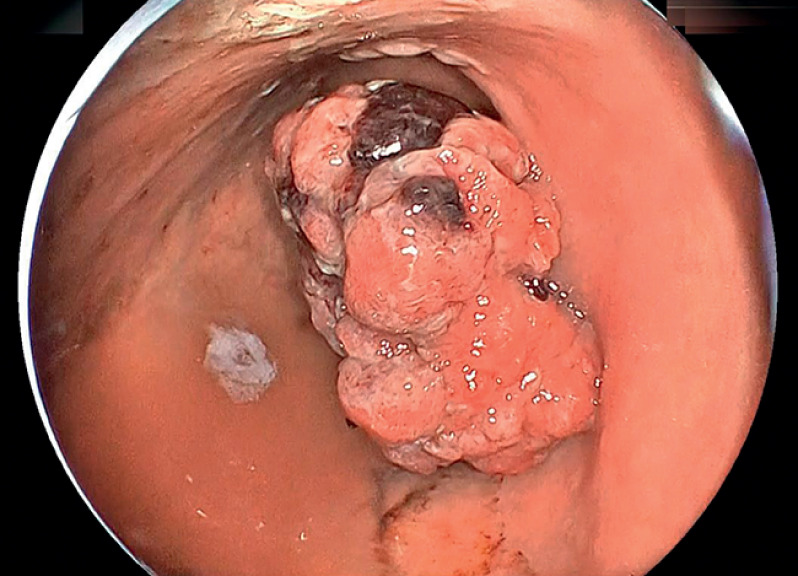
The lesion consisted of a 60-mm large type 0-Is with a 30-mm 0-IIa extension on the posterior wall of the lower gastric body.

**Fig. 2:**
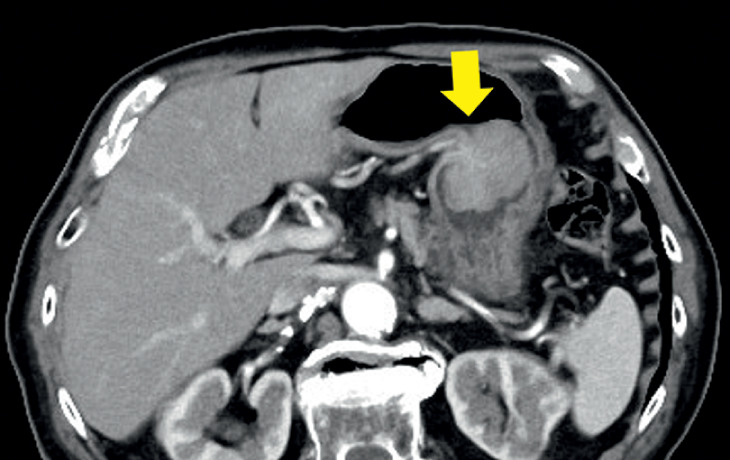
Enhanced computed tomography revealed that the muscle layer and a thick blood vessel were retracted into the large tumor.

Yet another advantage of saline-immersion therapeutic endoscopy!Video 1

Safe division of a resected lesion with a monopolar snare requires broad contact of the specimen with the gastric wall ( Fig. 3 ). Failure to achieve broad contact may result in heightened current density concentration at the smaller contact area rather than at the snare-constricted portion; this may cause failure of division, with potential deep-tissue injury and perforation at the smaller contact point ( Fig. 4 ) 5 . Through complete saline immersion, electrical conductivity of the medium facilitated electrical contact of the entire specimen with the gastric wall, enabling successful, rapid, safe division and retrieval without any adverse event ( Fig. 5 ).

**Fig. 3:**
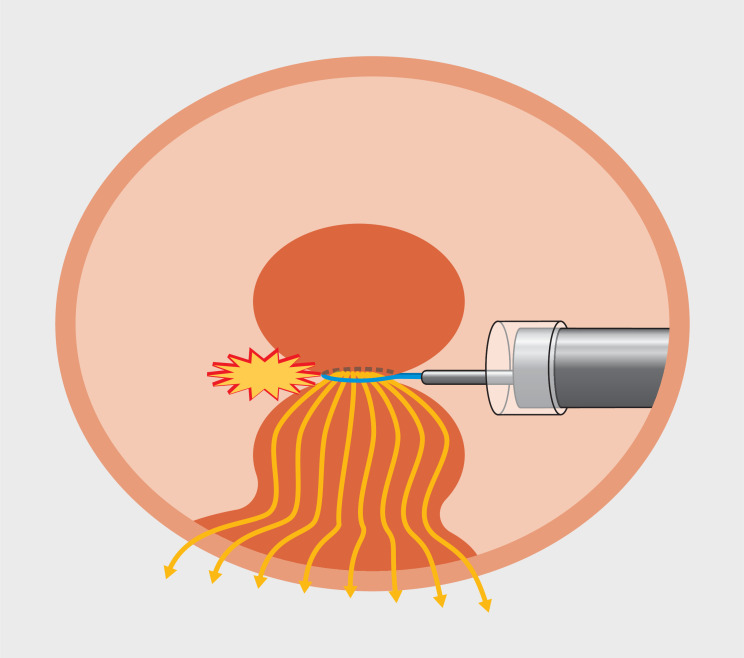
The current density concentrates at the portion constricted by the snare.

**Fig. 4:**
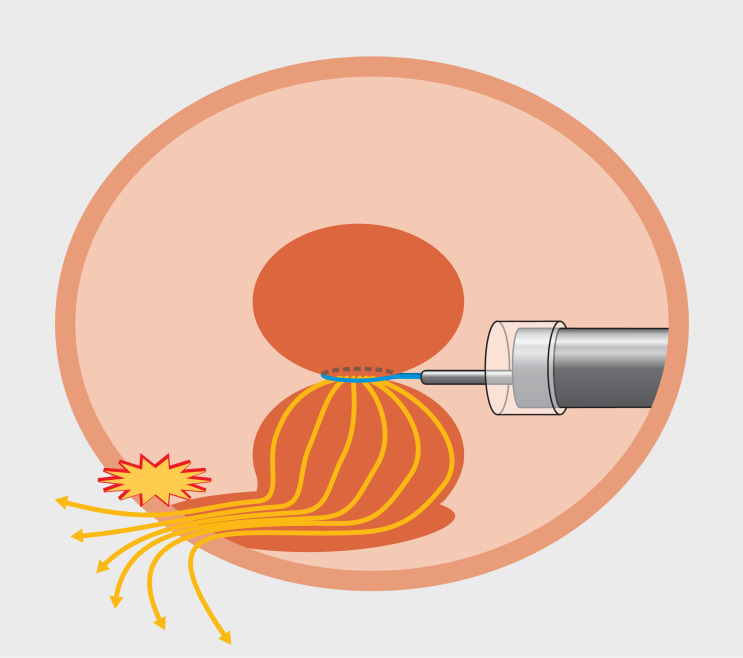
Current density concentration at a smaller contact area than the constricted portion, potentially causing ineffective cutting at the constricted portion with potential deep tissue injury and perforation at the contact point.

**Fig. 5:**
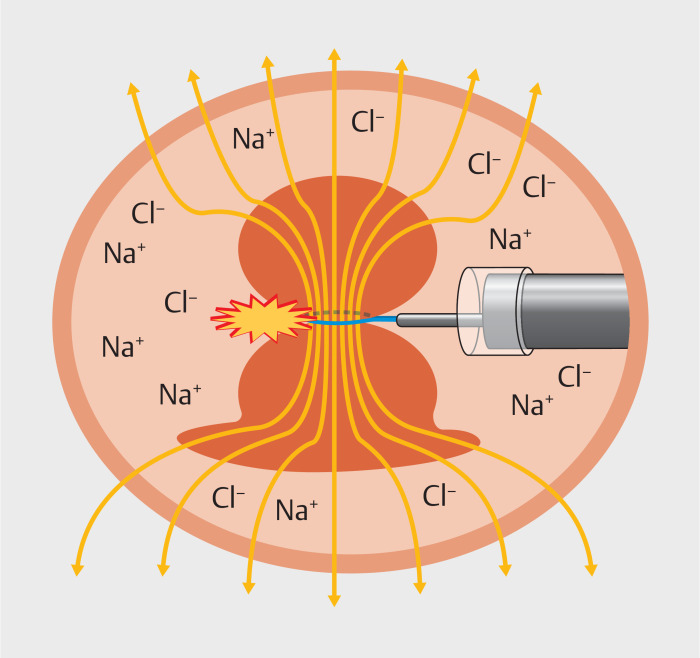
Through complete saline immersion of the resected specimen, conductivity of the medium allows for the entire specimen to maintain electrical contact with the gastric wall.

The advantages of SITE-facilitated PCM allowed safe management of fibrosis and thick vessels. Additionally, we highlight a further advantage of SITE: its efficacy for division of a bulky specimen using a monopolar snare for safe retrieval.

Endoscopy_UCTN_Code_TTT_1AO_2AG_3AD

## Notes

Endoscopy E-Videos https://eref.thieme.de/e-videos
*E-Videos* is an open access online section of the journal *Endoscopy* , reporting on interesting cases and new techniques in gastroenterological endoscopy. All papers include a high-quality video and are published with a Creative Commons CC-BY license. Endoscopy E-Videos qualify for HINARI discounts and waivers and eligibility is automatically checked during the submission process. We grant 100% waivers to articles whose corresponding authors are based in Group A countries and 50% waivers to those who are based in Group B countries as classified by Research4Life (see: https://www.research4life.org/access/eligibility/ ). This section has its own submission website at https://mc.manuscriptcentral.com/e-videos .
